# Genetic requirements for infection-specific responses in conferring disease resistance in *Arabidopsis*

**DOI:** 10.3389/fpls.2022.1068438

**Published:** 2022-11-29

**Authors:** Sung-Je Yoo, Hyo Ju Choi, Seong Woo Noh, Nicolás M. Cecchini, Jean T. Greenberg, Ho Won Jung

**Affiliations:** ^1^ Department of Molecular Genetics, Dong-A University, Busan, South Korea; ^2^ Department of Applied Bioscience, Dong-A University, Busan, South Korea; ^3^ Departamento de Química Biológica Ranwel Caputto, Centro de Investigaciones en Química Biológica de Córdoba (CIQUIBIC), Consejo Nacional de Investigaciones Científicas y Técnicas (CONICET), Universidad Nacional de Córdoba, Córdoba, Argentina; ^4^ Department of Molecular Genetics and Cell Biology, The University of Chicago, Chicago, IL, United States

**Keywords:** *Arabidopsis ALD1*, avirulent *Pseudomonas*, plant immune response, petiole exudates, salicylic acid

## Abstract

Immunity in plants arises from defense regulatory circuits that can be conceptualized as modules. Both the types (and isolates) of pathogen and the repertoire of plant receptors may cause different modules to be activated and affect the magnitude of activation. Two major defense enzymes of *Arabidopsis* are ALD1 and ICS1/SID2. ALD1 is an aminotransferase needed for producing the metabolites pipecolic acid, hydroxy-pipecolic acid, and possibly other defense signals. ICS1/SID2 produces isochorismate, an intermediate in the synthesis of salicylic acid (SA) and SA-derivatives. Metabolites resulting from the activation of these enzymes are found in petiole exudates and may serve as priming signals for systemic disease resistance in *Arabidopsis*. Mutants lacking ALD1 are known to have reduced SA accumulation. To further investigate the role of ALD1 in relation to the SA-related module, immunity phenotypes of double mutants that disrupt ALD1 and ICS1/SID2 or SA perception by NPR1 were compared with each single mutant after infection by different *Pseudomonas* strains. Exudates collected from these mutants after infection were also evaluated for their ability to confer disease resistance when applied to wild-type plants. During infection with virulent or attenuated strains, the loss of ALD1 does not increase the susceptibility of *npr1* or *sid2* mutants, suggesting the main role of ALD1 in this context is in amplifying the SA-related module. In contrast, after an infection that leads to strong pathogen recognition *via* the cytoplasmic immune receptor RPS2, ALD1 acts additively with both NPR1 and ICS1/SID2 to suppress pathogen growth. The additive effects are observed in early basal defense responses as well as SA-related events. Thus, there are specific conditions that dictate whether the modules independently contribute to immunity to provide additive protection during infection. In the exudate experiments, intact NPR1 and ICS1/SID2, but not ALD1 in the donor plants were needed for conferring immunity. Mixing exudates showed that loss of SID2 yields exudates that suppress active exudates from wild-type or *ald1* plants. This indicates that ICS1/SID2 may not only lead to positive defense signals, but also prevent a suppressive signal(s).

## Introduction

Plants have multilayered barriers to defend themselves against pathogen attacks. In the last few decades, plant immune receptors that initiate disease resistance against different infectious agents have been identified in model and crop plants (Han and Jung, 2013; Michelmore et al., 2013; Macho and Zipfel, 2014). Receptors that control resistance to bacterial pathogens mostly fall into two classes: cell surface-localized pattern recognition receptor (PRR) and cytoplasmic disease resistance (R) protein (also known as NOD-LIKE RECEPTOR [NLR] and nucleotide-binding site [NBS]-Leucine-rich repeat [LRR] protein). PRRs recognize microbe/pathogen-associated molecular patterns (MAMPs/PAMPs) and initiate pattern-triggered immunity (PTI), whereas R proteins are responsible for effector-triggered immunity (ETI) through direct or indirect recognition of the pathogen-derived effectors (Chisholm et al., 2006; Jones and Dangl, 2006; Jones et al., 2016). In general, the execution of PTI is characterized by an early Ca^2+^ and oxidative burst, transient dual phosphorylation of MITOGEN-ACTIVATED PROTEIN KINASE3 (MPK3) and MPK6, and induction of MAMP-responsive genes such as *FLG22-INDUCED RECEPTOR-LIKE KINASE1* (*FRK1*) and *NDR1/HIN1-LIKE10* (*NHL10*) (Nicaise et al., 2009; Serrano et al., 2007; Tsuda et al., 2013; Bigeard et al., 2015). In addition, salicylic acid (SA), a phytohormone critical for plant immunity to (hemi)biotrophic microbes, controls PRRs’ levels and intensifies the responsiveness of plants to MAMPs/PAMPs (Tateda et al., 2014). On the other hand, the most well-studied attribute of ETI is a hypersensitive response (HR), a type of programmed cell death occurring at infection sites (Mur et al., 2008). HR is accompanied by diverse cellular responses such as rapid ion leakage, chloroplast disruption, and DNA laddering (Coll et al., 2011). Various types of infections and/or MAMP treatments can have non-autonomous signaling effects that cause distal tissues to become more resistant to subsequent infections, a process called systemic acquired resistance (SAR) (Ryals et al., 1996; Mishina and Zeier, 2006; 2007; Zeidler et al., 2010; Jelenska et al., 2017; Cecchini et al., 2019).

Although PTI and ETI are initiated by different receptor systems, they share key defense components (Katagiri and Tsuda, 2010; Tsuda et al., 2013). Moreover, recent studies demonstrate that ETI enhances PTI and requires PTI for full ETI against avirulent *Pseudomonas* infection (Ngou et al., 2021; Yuan et al., 2021). However, there are still many questions about the relationship between PTI and ETI. One of the shared defense signals is SA, which is implicated in both PTI and ETI pathways (Glazebrook, 2005; Spoel and Dong, 2008; Tsuda et al., 2013; Tateda et al., 2014). In addition, SA is needed for the establishment of SAR (Métraux et al., 1990; Durrant and Dong, 2004). Dozens of proteins bring SA-dependent defenses to completion. Among them, the crucial defense factors, ICS1/SID2, NPR1, and ALD1, are important for the interconnectivity between PTI, ETI and a SA-dependent defense response. ISOCHORISMATE SYNTHASE1/SALICYLIC ACID INDUCTION DEFICIENT2 (ICS1/SID2) is directly engaged in SA biosynthesis (Nawrath and Métraux, 1999; Wildermuth et al., 2001), whereas NONEXPRESSOR OF PATHOGENESIS-RELATED PROTEINS1 (NPR1), a SA receptor, is necessary for the expression of SA-responsive genes (Cao et al., 1997; Fu et al., 2012; Wu et al., 2012). AGD2-LIKE DEFENSE PROTEIN1 (ALD1), a diaminopimelate aminotransferase, is responsible for early SA accumulation during ETI as well as for the biosynthesis of pipecolic acid (Pip)/N-hydroxy-Pip (NHP) during infections that lead to SAR (Song et al., 2004a; 2004b; Návarová et al., 2012; Sobolev et al., 2013; Hartmann et al., 2017; 2018; Chen et al., 2018; Jiang et al., 2021). ALD1 is also involved in PTI and the basal production of unidentified non-Pip molecule(s) that induces/maintains local disease resistance, but not systemic resistance (Cecchini et al., 2015). Pip, NHP, and other non-Pip molecules are proposed to be present in the petiole exudates of leaves infected with SAR-inducing *Pseudomonas* (Návarová et al., 2012; Chen et al., 2018; Hartmann et al., 2018; Jiang et al., 2021). Moreover, both ALD1 and ICS1/SID2 are required for the accumulation of the receptor for the MAMP flagellin, FLAGELLIN-SENSING2 (FLS2), in *Arabidopsis*, and NPR1 participates in the flg22 (a flagellin-derived peptide that triggers FLS2 signaling)-induced early immune response (Cecchini et al., 2015; Yi et al., 2014; 2015).

Two well-known R proteins activating strong ETI in *Arabidopsis*are the cytoplasmic receptors RESISTANCE TO PSEUDOMONAS SYRINGAE2 (RPS2) and RESISTANCE TO P. SYRINGAE PV MACULICOLA1 (RPM1). RPS2 and RPM1 confer resistance against infection by avirulent *Pseudomonas syringae* carrying *AvrRpt2* and *AvrRpm1*, respectively (Kunkel et al., 1993; Bisgrove et al., 1994). Although RPS2- and RPM1-mediated immunity require common genes (*e.g.*, NON-RACE-SPECIFIC DISEASE RESISTANCE1 [NDR1]), RPS2 signaling results in stronger SA-related defenses upon infection (Century et al., 1995; Belkhadir et al., 2004; Day et al., 2006; Lee et al., 2007). Avirulent *P. syringae* derivatives grow better, but cause weak or no detectable visible symptoms in leaves of *ald1*, *npr1*, and *sid2* mutants, compared with wild type (WT), when the inoculum concentrations are low (*e.g.*, OD_600_ = 0.0001) (Nawrath and Métraux, 1999; Clarke et al., 2000; Song et al., 2004b; Raffaele et al., 2006; Venugopal et al., 2009). Additionally, macroscopic HR-associated leaf collapse still occurs in these mutants, although NPR1 and ICS1/SID2 somewhat affect the development of HR symptoms after infection with avirulent strains of *P. syringae* (> OD_600_ = 0.01). (Nawrath and Métraux, 1999; Dewdney et al., 2000; Rate and Greenberg, 2001; Song et al., 2004b; Zhang et al., 2004; Munch et al., 2014). Microscopic early cell death response after similar avirulent *P. syringae* infections is highly reduced in *sid2* plants (Seguel et al., 2018). These previous observations show that ALD1, NPR1, and ICS1/SID2 are engaged in R-mediated immunity as well as SA-dependent defense when avirulent pathogens infect *Arabidopsis* plants.

Although several genetic studies exist (Lu et al., 2009; Ng et al., 2011), the importance of different defense modules mediated by ALD1 and NPR1 or ICS1/SID2 during PTI and ETI have yet to be fully elucidated. In particular, because of ALD1’s role in positively affecting SA levels, we wished to test whether ALD1 acts primarily by amplifying SA levels or if it has an additive function with SA synthesis and/or transduction. We addressed this gap in knowledge and found that there are different requirements for ALD1, NPR1 and ICS1/SID2, depending on the *P. syringae* strain used for infection. In many infections, ALD1’s role is to amplify SA, but one avirulent infection uncovered an additive function for ALD1 with SA. We infer that the receptors used for detecting different strains influence how the defense network is modulated to achieve pathogen suppression. Finally, we also investigated the roles of ALD1, NPR1 and ICS1/SID2 in the production of active exudates that may contain mobile defense signals. We find different roles for these components in the production of active exudates and discuss the implications of these findings for understanding systemic signaling.

## Materials and methods

### Biological materials

*A. thaliana* seeds treated with 95% ethanol for 2 ~ 3 min were soaked in 50% bleach supplemented with 0.01% Triton X-100 for 15 min. The seeds were washed with deionized water several times to remove residual bleach. For most experiments (except those involving exudates-see section below), the resulting sterilized seeds were sown in potting mixture consisting of 59.26% peat moss, 20% cocopeat, and 20% perlite (Nongwoo Bio, Korea). Plants grew from 24 to 28 days in an environmentally controlled walk-in growth chamber (21 ± 1°C, 12 h day and 12 h night, 50 to 60% humidity). All seeds were in the Columbia (Col-0) background. To generate double mutants, *ald1-T2* (*ald1*) was crossed with *npr1-1* (*npr1*) or *sid2-1* (*sid2*), and the resulting mutants were screened with T-DNA verification primers (*ald1*) and dCAPS markers (*npr1* and *sid2*) (**Table 1**) (Cao et al., 1997; Nawrath and Métraux, 1999; Song et al., 2004a).

**Table 1:** Nucleotide sequences of primers used in this study.

Usage	Gene	Forward primer (5’➔3’)	Reverse primer (5’➔3’)
qRT-PCR	*ACTIN2*	AGTGTCTGGATCGGTGGTTC	CCCCAGCTTTTTAAGCCTTT
	*FRK1*	GCCAACGGAGACATTAGAG	CCATAACGACCTGACTCATC
	*NHL10*	TTCCTGTCCGTAACCCAAAC	CCCTCGTAGTAGGCATGAGC
	*PR1*	AAGGCCCACCAGAGTGTATG	TTCTTCCCTCGAAAGCTCAA
Genotyping	ALD1	TTGCTCTGGAATAGGCTCTGT	AGTAAAGAATGGTCAGTCTAATG
	*ald1-T2*	TTGCTCTGGAATAGGCTCTGT	GCGTGGACCGCTTGCTGCAACT
	*npr1-1*	GTTAGTCTTGAAAAGTCATTGCCGGAAG	TTTCGGCGATCTCCATTGCAGC
	*sid2-1*	AATCAAAAGCCTTCTTC	CATTTCTTGGATAATAGTTTGG

Two virulent strains, *P. syringae* pv. *maculicola* ES4326 (re-classified as *P. cannabina* pv. *alisalensis* [Bull et al., 2010]) (*Psm*ES4326) and *P. syringae* pv. *tomato* DC3000 (*Pst*DC3000), and an attenuated strain *Pst*DC3000 *ΔAvrPto*/*ΔAvrPtoB* (Lin and Martin, 2005) were employed in this study. Four different previously described avirulent derivatives of *Psm*ES4326 and *Pst*DC3000 carrying *AvrRpt2* or *AvrRpm1* were used (Mudgett and Staskawicz, 1999; Guttman and Greenberg, 2001).

### Pathogen infection

*Pseudomonas* strains were cultured on King’s B (KB) media (10 g proteose peptone, 1.5 g K_2_HPO_4_, 15 g glycerol, and 5 mM MgSO_4_ per liter) supplemented with an appropriate antibiotic(s) at 28°C. Freshly cultured *Pseudomonas* strains were diluted to an optical density at 600 nm (OD_600_) = 0.0001, 0.001 or 0.1 in 10 mM MgSO_4_. Bacterial suspensions diluted to OD_600_ = 0.001 or 0.0001 were infiltrated into the leaves of WT and mutants with a needleless syringe. Infected plants were covered with a transparent plastic dome to keep high relative humidity for successful disease development. For spray-inoculation, 0.05% Silwet^®^ L-77 was added into the bacterial suspension (OD_600_ = 0.1) before application.

The number of bacteria was determined using serial-dilutions by plating on KB media on day 3 (syringe-inoculation) or 5 (spray-inoculation) after infection. To monitor visible HR formation in the WT and mutants induced by either *Psm*ES4326/*AvrRpt2* or *Psm*ES4326/*AvrRpm1*, 3 leaves per plant were infected by a high dose of these avirulent strains (OD_600_ = 0.01) and the number of leaves showing visible or no HR was scored 10 h (for RPM1-AvrRpm1 interaction) and 20 h (for RPS2-AvrRpt2 interaction) after inoculation.

### Free SA measurement and electrolyte leakage analysis

Free SA levels were quantified as described with minor modifications (Seskar et al., 1998; Jung et al., 2009). An avirulent derivative of *Pseudomonas, Psm*ES4326/*AvrRpt2* (OD_600_ = 0.01), was infiltrated into the leaves of *Arabidopsis* plants. The infected leaves were harvested at given time points to check the level of free SA induced by pathogen infection. *o*-anisic acid (W394300, Sigma-Aldrich) was spiked into the samples before extraction to calibrate the recovery rate after extraction and HPLC analysis. Extracts were dried, dissolved in 55% methanol and applied in 10 μl aliquots to an HPLC coupled with a fluorescence detector (Agilent 1100). The experiments were repeated twice with similar results.

The degree of electrolyte leakage was measured in the leaves of WT and mutants infected with either *Psm*ES4326/*AvrRpt2* or *Psm*ES4326/*AvrRpm1* (OD_600_ = 0.05) until 25 h after inoculation. Ten leaf discs per biological replicate (4 replicates) were taken from the infected leaves, rinsed with distilled water for 30 min, and submerged in distilled water (Sohn et al., 2012). The conductivity was measured with a LAQUAtwin compact water quality metre (Horiba Scientific).

### Real-time quantitative reverse transcription PCR analysis

Bacterial strains employed in this study were infiltrated into the leaves of *Arabidopsis* (OD_600_ = 0.01). Plants were kept in a walk-in growth chamber without a plastic dome during infection. Total RNAs from the infected *Arabidopsis* leaves were isolated with the TRIzol™ reagent according to the manufacturer’s instruction (Thermo Fisher Scientific). In order to minimize genomic DNA contamination in total RNAs, TURBO™ DNase (Thermo Fisher Scientific) was added to the total RNAs at 37°C for 30 min. About 1 μg of total RNA was used to synthesize first-strand cDNAs with Superscript™ II reverse transcriptase (Thermo Fisher Scientific). qRT-PCR was performed with the SYBR™ Green PCR mixture (Takara Bio) using the cycling program as follows: 95°C for 5 min followed by 40 cycles at 95°C for 15 s, 60°C for 15 s, and 72°C for 35 s (CFX384™/C1000™, Bio-Rad). *ACTIN2* (At3g18780) was used as an internal control. Oligonucleotide sequences used in this study were listed in **Table 1**. Relative expression levels were calculated by a relative standard curve method (Larionov et al., 2005). The mRNA analyses were repeated at least three times with similar expression patterns.

### Western blot analysis

Leaves of *Arabidopsis* plants inoculated with 10 mM MgSO_4_, *Pst*DC3000, *Pst*DC3000 *ΔAvrPto*/*ΔAvrPtoB*, and *Psm*ES4326*/AvrRpt2* (OD_600_ = 0.01) were collected at indicated time points. The finely ground leaf powder was homogenized in an equal volume of protein extraction buffer (20 mM Tris-HCl [pH 7.5], 150 mM NaCl, 1 mM EDTA, 1% Triton X-100, 0.1% sodium dodecyl sulfate [SDS], 5 mM dithiothreitol, and 1 X proteinase inhibitor [Thermo Fisher Scientific]) and centrifuged at 16,200 x *g* for 30 min in order to eliminate certain debris (Lee et al., 2007). SDS-polyacrylamide gel electrophoresis (SDS-PAGE) using Tris-glycine electrophoresis buffer was carried out as described by Sambrook et al. (1989). Target proteins and concentration of primary antibodies were used in this study as follows: Phospho-P44/P42 MAPK (Erk1/2) (#4370L, Cell Signaling Technology), 1:1000; MPK3 (M8318, Sigma-Aldrich), 1:800; MPK6 (A7104, Sigma-Aldrich), 1:5000; and BAK1 (AS12 1858, Agrisera), 1:5000. A goat anti-rabbit immunoglobulin G (H+L) conjugated with horseradish peroxidase (Thermo Fisher Scientific) was employed as secondary antibodies (1:5000). The signal was visualized with SuperSignal™ Chemiluminescent Substrate (Thermo Fisher Scientific). All immunoblot analyses were repeated at least twice, and the relative band intensities against a Coomassie Brilliant Blue (CBB) staining were quantified using the Image J program.

### Petiole exudate analysis

WT and mutant plants were grown on Pro-Mix BX (Premier Tech) soil for 24 ~ 28 days under 21 ± 1°C and 12 h day/12 h night conditions (Greenberg et al., 2000). Leaves of *A. thaliana* were inoculated with either 10 mM MgSO_4_ or *Psm*ES4326/*AvrRpt2* (OD_600_ = 0.01) and detached 12 to 15 h after inoculation. To collect exudates, leaves were surface sterilized with 70% ethanol, and petioles were submerged into 1 mM EDTA supplemented with 50 μg/ml carbenicillin and streptomycin (Duchefa) at room temperature under continuous light as described (Jung et al., 2009). Exudates were diluted five times in 10 mM MgSO_4_ and stored at -80°C. For treatments with mixed exudates, we added an equal volume of 10 mM MgSO_4_ or *sid2*-exudates into WT-exudates or *ald1*-exudates. To test whether or not petiole exudates could confer resistance to bacterial infection in *Arabidopsis*, diluted petiole exudates were syringe-infused into recipient WT leaves 2 days prior to challenge-inoculation with virulent *Psm*ES4326 (OD_600_ = 0.0001) (Jung et al., 2009). Bacterial growth was enumerated on day 3 after inoculation.

## Results

### Loss of ALD1 does not make *npr1* and *sid2* mutants more susceptible to infection with virulent *Pseudomonas*

**Figure 1:**
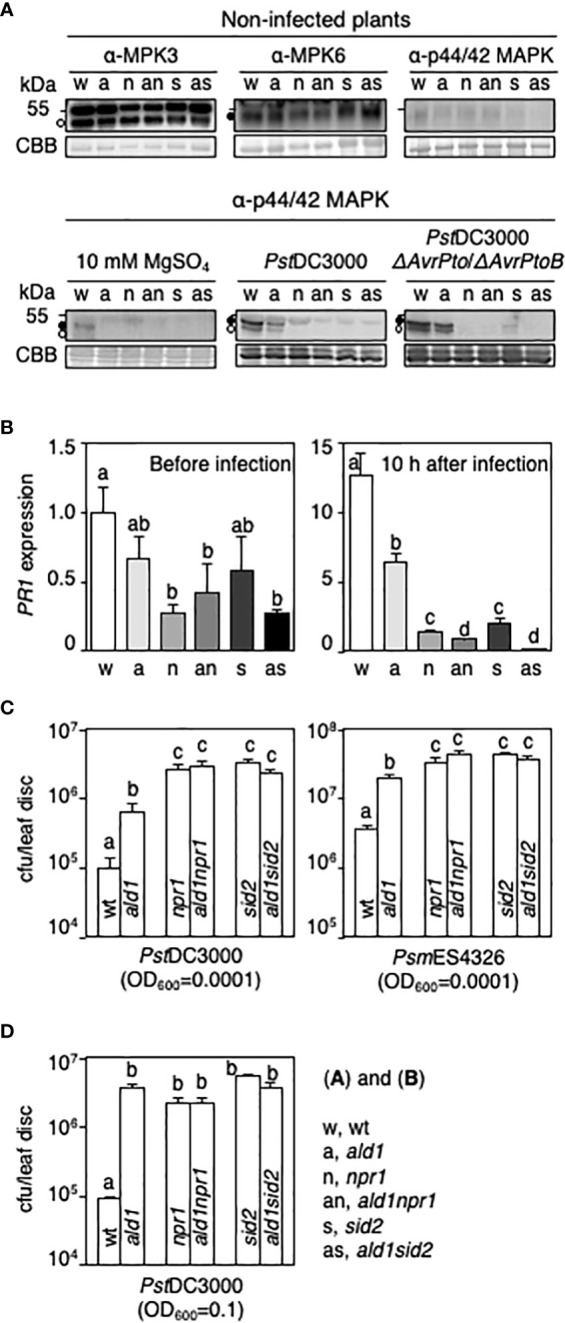
Loss of ALD1 in *npr1* or *sid2* mutants does not alter the susceptibility of *Arabidopsis* against infection by virulent strains of *Pseudomonas*. **(A)** Levels of MPK3, MPK6, and phosphorylated MPK3 and MPK6 in leaves of WT (w), *ald1* (a), *npr1* (n), *ald1npr1* (an), *sid2* (s), and *ald1sid2* (as) without (upper panel) and with pathogen infection (lower panel). Open circles and filled circles point out MPK3 and MPK6, respectively. Membranes were stained with Coomassie Brilliant Blue R-250 (CBB) to verify equal loading. Infected leaves with 10 mM MgSO4, *Pst*DC3000, or *Pst*DC3000 *AvrPto*/*AvrPtoB* (OD_600_ = 0.01) were taken 30 min after infiltration. **(B)** Relative *PR1* mRNA level in leaves of WT and mutants during *Psm*ES4326 infection (OD_600_ = 0.01). mRNA level in the non-infected WT plant (*PR1*/*ACTIN2*) was set to 1, and then mRNA levels of each condition were normalized by the value of the non-infected WT plant. Error bars represent the standard deviation from three biological replicates (number of technical replicates = 3). **(C)** Growth of virulent strains of *Pseudomonas* in *Arabidopsis* plants 3 days after syringe-inoculation. Leaves of 24- to 28-day-old plants were inoculated with either a virulent strain of *Pst*DC3000 (left panel) or *Psm*ES4326 (right panel) (OD_600_ = 0.0001) by using a needleless syringe. **(D)** Growth of *Pst*DC3000 in leaves of *Arabidopsis* plants 5 days after spray-inoculation (OD_600_ = 0.1). Error bars indicate standard error (n = 8 for syringe-inoculation; n = 12 for spray-inoculation) **(C, D)**. The experiments were repeated more than three times with the same results. Different letters above bars indicate statistically significant differences (*p* < 0.05, one-way ANOVA) **(B-D)**.

Mutation of *NPR1*, but not *ICS1/SID2*, was reported to adversely affect dual phosphorylation of MPK3 and MPK6 in *Arabidopsis* after infection with *P. syringae* or flg22 treatment (Tsuda et al., 2013; Yi et al., 2015). To test if ALD1 cooperates with NPR1 and ICS1/SID2 for establishing early basal defense, we first monitored levels of MPK3 and MPK6 and the amount of activated MPK3 and MPK6 (phosphorylated forms) in leaves of WT, *ald1*, *npr1*, *sid2*, *ald1npr1*, and *ald1sid2* plants before and after infection with a virulent or an attenuated strain of *P. syringae*. We used the attenuated strain *Pst*DC3000 *ΔAvrPto*/*ΔAvrPtoB,* because the AvrPto and AvrPtoB secreted virulence effectors inhibit early basal defense responses initiated by MAMP-perception as well as the level of PRR complexes in *Arabidopsis* (Block and Alfano, 2011). Untreated plants showed no changes in the total MPK3 and MPK6 levels (upper panel in **Figure 1A**), indicating that basal accumulation of MPK3 and MPK6 occurs independently of ALD1, NPR1, and SID2. Phosphorylated MPK3 and MPK6 levels were very low in untreated WT and all of the mutants (upper panel in **Figure 1A**). Infection with *Pst*DC3000 or *Pst*DC3000 *ΔAvrPto*/*ΔAvrPtoB* led to robust phosphorylation of MPK3 and MPK6 in WT 30 min after infection (lower panel in **Figure 1A**). The extent of phosphorylated MPK3 and MPK6 was reduced in the *ald1* mutant as compared with that in the WT. In *npr1*, *sid2*, *ald1npr1*, and *ald1sid2* plants, no phosphorylated MPK3 or MPK6 was detected (lower panel in **Figure 1A**). Mock inoculation with 10 mM MgSO_4_ only slightly activated MPK3 and MPK6 in WT 30 min after infiltration. These results suggest that ALD1 is only partially needed, whereas NPR1 and SID2 are strictly required for the phosphorylated-forms to accumulate at 30 min after *Pst*DC3000 *ΔAvrPto*/*ΔAvrPtoB* or *Pst*DC3000 inoculation. ALD1 may influence the transient phosphorylation of MPK3 and MPK6 in NPR1- and ICS1-dependent manners. The levels of phosphorylated MPK3 and MPK6 observed in *npr1* and *sid2* mutants in this study differ from those previously reported (Tsuda et al., 2013; Yi et al., 2015). This might be due to the non-sterile growth conditions used here and differences in the time points used among experiments done in different labs.

To test whether SA-responsive defenses are reduced in the double mutants compared to each single mutant, we examined the expression of *PATHOGENESIS-RELATED PROTEIN1* (*PR1*) in leaves of *Arabidopsis* with or without pathogen infection. Under uninfected conditions, the level of *PR1* transcript was diminished in *npr1*, *ald1npr1*, and *ald1sid2*, as compared with those in WT (left panel in **Figure 1B**). Mutation of *NPR1* or *ICS1*/*SID2* strongly decreased *PR1* transcription more than that of *ALD1* during *Psm*ES4326 infection (OD_600_ = 0.01) (right panel in **Figure 1B**). Interestingly, *PR1* levels in *ald1npr1* and *ald1sid2* were lower than those in *npr1* and *sid2* mutants 10 h after inoculation (right panel in **Figure 1B**). Considering the additive effect between ALD1 and NPR1 or ICS1/SID2, the results strongly support that ALD1 can impact *PR1* expression during infection in a SA- and NPR1-independent manner as described previously using a constitutive defense mutant (Song et al., 2004b; Ng et al., 2011).

To analyze if the changes in MPK3 and MPK6 activation and *PR1* transcripts by the simultaneous mutation of ALD1, NPR1 and/or ICS1 are related to resistance against pathogens, we syringe-inoculated *Arabidopsis* plants with virulent strains of *Pseudomonas* and measured bacterial growth in infected leaves 3 days post inoculation. The *ald1* mutant was less susceptible than *npr1* and *sid2* mutants to *Pst*DC3000 and *Psm*ES4326 (**Figure 1C**). The mutation of *ALD1* in the *npr1* or *sid2* background did not affect the susceptibility to either strain (**Figure 1C**). These results suggest that the residual resistance in *ald1* is due to the SA pathway regulated by NPR1 and ICS1/SID2, at least when bacteria is infiltrated.

Epidermal cells provide a substantial barrier to infection, possibly due to the cuticle and the dynamics of natural openings such as stomata. Moreover, ALD1 restricted to the epidermal plastids rescues several immune responses in *Arabidopsis* (Jiang et al., 2021). One way to assess the role of epidermal defenses is to spray-inoculate bacteria onto plant leaves and measure bacterial growth (Melotto et al., 2006; Panchal et al., 2016). Therefore, we spray-inoculated WT and our suite of *Arabidopsis* mutants with *Pst*DC3000. The *ald1* plants showed more growth of bacteria relative to that seen in WT. Additionally, *ald1* susceptibility was indistinguishable from that of *npr1*, *sid2*, *ald1npr1*, and *ald1sid2* (**Figure 1D**). Thus, ALD1 and the SA biosynthesis/action components (ICS1/SID2 and NPR1) are needed together to suppress strong colonization after spray-inoculation, as suggested by a previous study (Jiang et al., 2021). This may be due to ALD1’s role in amplifying SA accumulation.

### Loss of ALD1 in *npr1* and *sid2* mutants does not additively increase susceptibility to infection with attenuated *Pseudomonas*

**Figure 2:**
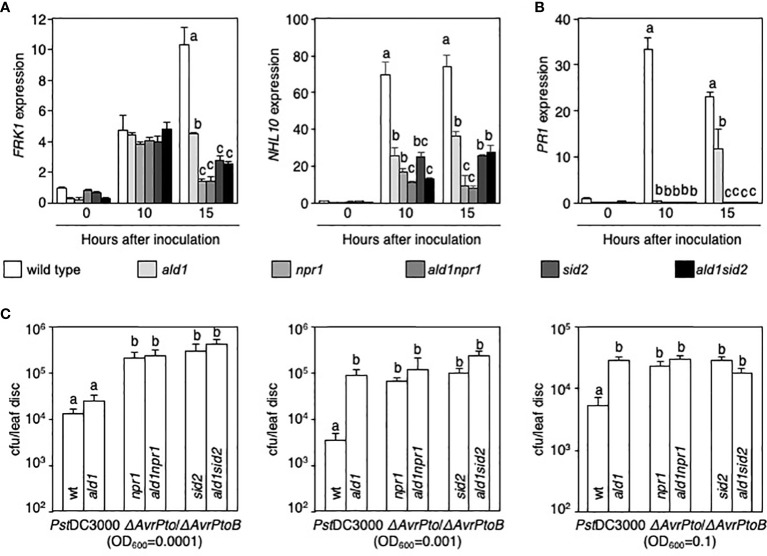
Mutations of *ALD1*, *NPR1*, and *ICS1*/*SID2* cause hypo-resistance against an attenuated strain of *Pseudomonas syringae* infection. **(A, B)** Relative expression levels of *FRK1* and *NHL10* **(A)** and *PR1* **(B)** in leaves of WT and mutants infected with *Pst*DC3000 *ΔAvrPto*/*ΔAvrPtoB* (OD_600_ = 0.01). The columns and bars show the average with standard deviation obtained from three biological replications (number of technical replicates = 3). **(C)** Growth of *Pst*DC3000 *ΔAvrPto*/*ΔAvrPtoB* strain in WT and mutants. An attenuated strain was syringe-infiltrated into the leaves of WT, single, and double mutants (OD_600_ = 0.0001 or 0.001) (left and middle panels), or the strain was also applied to *Arabidopsis* plants with a spray (OD_600_ = 0.1) (right panel). The number of bacteria was counted on day 3 or 5 after inoculation. Error bars indicate standard error (n = 10 or 12). The experiment was repeated twice with the same results. Statistically significant differences are shown using different letters (*p* < 0.05 [**A**, **B**], *p* < 0.01 [**C**], one-way ANOVA).

The reduced dual phosphorylation of MPK3 and MPK6 in single and double mutants 30 min after inoculation (**Figure 1A**) prompted us to test whether ALD1, NPR1, and ICS1/SID2 control basal defense responses to *Pst*DC3000 *ΔAvrPto*/*ΔAvrPtoB* infection. We analyzed transcript levels of *FRK1*, *NHL10*, and *PR1* in WT and mutants during infection with the attenuated *Pseudomonas* strain (Lin and Martin, 2005; Rosebrock et al., 2007; Boudsocq et al., 2010; Mersmann et al., 2010). Although differences in *FRK1* transcript levels were not found among WT and mutant plants 10 h after *Pst*DC3000 *ΔAvrPto*/*ΔAvrPtoB* inoculation, the level of *FRK1* mRNA significantly decreased in the mutants, especially *npr1*, *sid2*, *ald1npr1*, and *ald1sid2*, 15 h after inoculation (left panel in **Figure 2A**). Transcript levels of *NHL10*, another MAMP-responsive gene, decreased in all mutants tested in this study after *Pst*DC3000 *ΔAvrPto*/*ΔAvrPtoB* infection compared with that in WT. *NHL10* mRNA expression was also significantly reduced in double mutants compared with those in each single mutant 10 h after inoculation (right panel in **Figure 2A**). In the case of *PR1*, there was no induction of the gene in any of the mutants at 10 h and only the *ald1*mutant displayed an increase (although less than in WT) at 15 h after *Pst*DC3000 *ΔAvrPto*/*ΔAvrPtoB* infection (**Figure 2B**). Expression levels of *PR1* in the *ald1npr1* and *ald1sid2* mutants did not differ from those in *npr1* and s*id2* mutants after *Pst*DC3000 *ΔAvrPto*/*ΔAvrPtoB* infection (**Figure 2B**). This supports the view that NPR1 and ICS1/SID2 are the leading players in regulating *PR1* expression in *Arabidopsis* (Cao et al., 1997; Wildermuth et al., 2001). Taken together, these mRNA analyses reveal that NPR1 and ICS1/SID2 have a stronger influence on *FRK1*, *NHL10*, and *PR1* mRNA expression than ALD1 in *Pst*DC3000 *ΔAvrPto*/*ΔAvrPtoB-*infected leaves.

We analyzed the growth of the attenuated strain in WT and mutant plants. The bacterial titer was slightly higher in the *ald1* mutant than in WT when *Pst*DC3000 *ΔAvrPto*/*ΔAvrPtoB* (OD_600_ = 0.0001) were infiltrated into *Arabidopsis* leaves; however, the difference was not statistically significant (left panel in **Figure 2C**). Interestingly, relative to WT, *ald1* showed increased susceptibility to *Pst*DC3000 *ΔAvrPto*/*ΔAvrPtoB* infection with a higher concentration (OD_600_ = 0.001) (middle panel in **Figure 2C**) as well as after spray-inoculation of the strain (OD_600_ = 0.1) (right panel in **Figure 2C**). Populations of the attenuated strain were higher in *npr1* and *sid2* mutants than in the WT plant, and a further mutation of *ALD1* in *npr1* and *sid2* mutants did not render plants more susceptible to *Pst*DC3000 *ΔAvrPto*/*ΔAvrPtoB* infection (**Figure 2C**). This finding, together with the intermediate effect of mutation of *ALD1* on MPK3/MPK6 activation and defense genes expression supports the view that ALD1 amplifies the ICS1/SID2 and NPR1 module in this infection condition.

### Both *ald1npr1* and *ald1sid2* mutants are more susceptible than each single mutant to *Pseudomonas* carrying *AvrRpt2*, but not *AvrRpm1*

**Figure 3:**
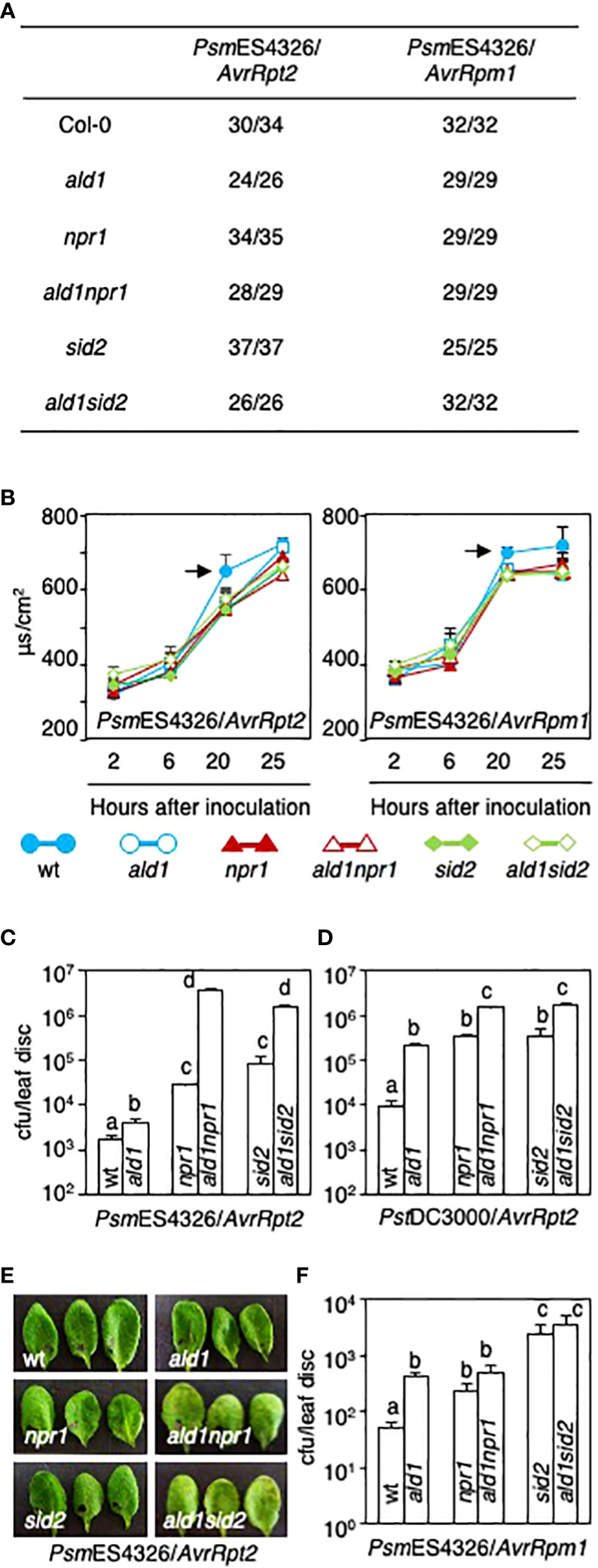
Both *ald1npr1* and *ald1sid2* mutants show susceptibility in response to avirulent derivatives of *Pseudomonas* carrying *AvrRpt2*, but not *AvrRpm1*. **(A)** The number of leaves exhibiting HR out of the number of leaves infected with two different avirulent derivatives, *Psm*ES4326/*AvrRpt2* or *Psm*ES4326/*AvrRpm1* (OD_600_ = 0.01). **(B)** Electrolyte leakage from leaves of *Arabidopsis* after infection with *Psm*ES4326/*AvrRpt2* (left panel) or *Psm*ES4326/*AvrRpm1* (right panel) (OD_600_ = 0.05). Data represent the averages with standard deviations (n = 4). Arrows indicate statistically significant differences between WT and mutants (*p* < 0.01, two-tailed Student’s t-test). The experiments were repeated twice with the same results. **(C, D)** Pathogen growth in leaves of *ald1npr1* and *ald1sid2* mutants after *Psm*ES4326/*AvrRpt2* infection **(C)** and *Pst*DC3000/*AvrRpt2* infection (OD_600_ = 0.0001) **(D)**. **(E)** Symptom development in the leaves of *ald1npr1* and *ald1sid2* mutants after *Psm*ES4326/*AvrRpt2* infection (OD_600_ = 0.0001). The photos were taken on day 3 after inoculation. **(F)** Growth of an avirulent derivative, *Psm*ES4326/*AvrRpm1*, in leaves of *Arabidopsis* 3 days after inoculation (OD_600_ = 0.0001). Data **(C, D, F)** represent the averages with standard errors (n = 8, or 12). Different letters indicate statistically significant differences (*p* < 0.01, one-way ANOVA). The experiments were repeated more than three times with the same results.

To examine if disrupting ALD1 and NPR1 or ICS1/SID2 might have an additive effect on ETI in *Arabidopsis*, we inoculated avirulent derivatives of *Psm*ES4326 and *Pst*DC3000 carrying *AvrRpt2* or *AvrRpm1* into leaves of WT and mutant plants. Although visible HR was not affected by any mutations tested in this study after avirulent *Pseudomonas* infection (OD_600_ = 0.01), the rate of electrolyte leakage was slightly delayed by these mutations (**Figures 3A, B**). Previously, it was reported that the *npr1* mutant showed stronger HR than the WT after infection with *P. syringae* carrying *AvrRpm1* (OD_600_ = 0.03 or 0.1), but exhibited a WT-like response to *P. syringae* carrying *AvrRpt2* (OD_600_ = 0.03) (Rate and Greenberg, 2001). These current and previous studies suggest that the cell death response of *npr1* partly relies on the concentration of inoculum and the genetic composition of *P. syringae*.

Interestingly, the growth of *Psm*ES4326/*AvrRpt2* and *Pst*DC3000/*AvrRpt2* dramatically increased more than 10-fold in the *ald1npr1* and *ald1sid2* mutants compared with those in each single mutant (**Figures 3C, D** **)**. Additionally, the double mutants exhibited visible disease symptoms after *Psm*ES4326/*AvrRpt2* infection, while no evident disease lesions in WT or single mutants were observed (**Figure 3E**). In contrast to the effects of strains carrying *AvrRpt2*, growth of *Psm*ES4326/*AvrRpm1* did not increase in the *ald1npr1* and *ald1sid2* mutants compared to that in each single mutant (**Figure 3F**). These results show that effective signaling *via* RPS2, the AvrRpt2 receptor, requires more than just an amplifying effect of ALD1 on SA accumulation previously described (Song et al., 2004b). Thus, we suggest that ALD1 provides additional SA-independent defenses for suppressing the growth of strains that carry AvrRpt2.

### ALD1 and NPR1 have an additive effect on SA and *PR1* accumulation against an avirulent derivative of *Pseudomonas* carrying *AvrRpt2*

**Figure 4:**
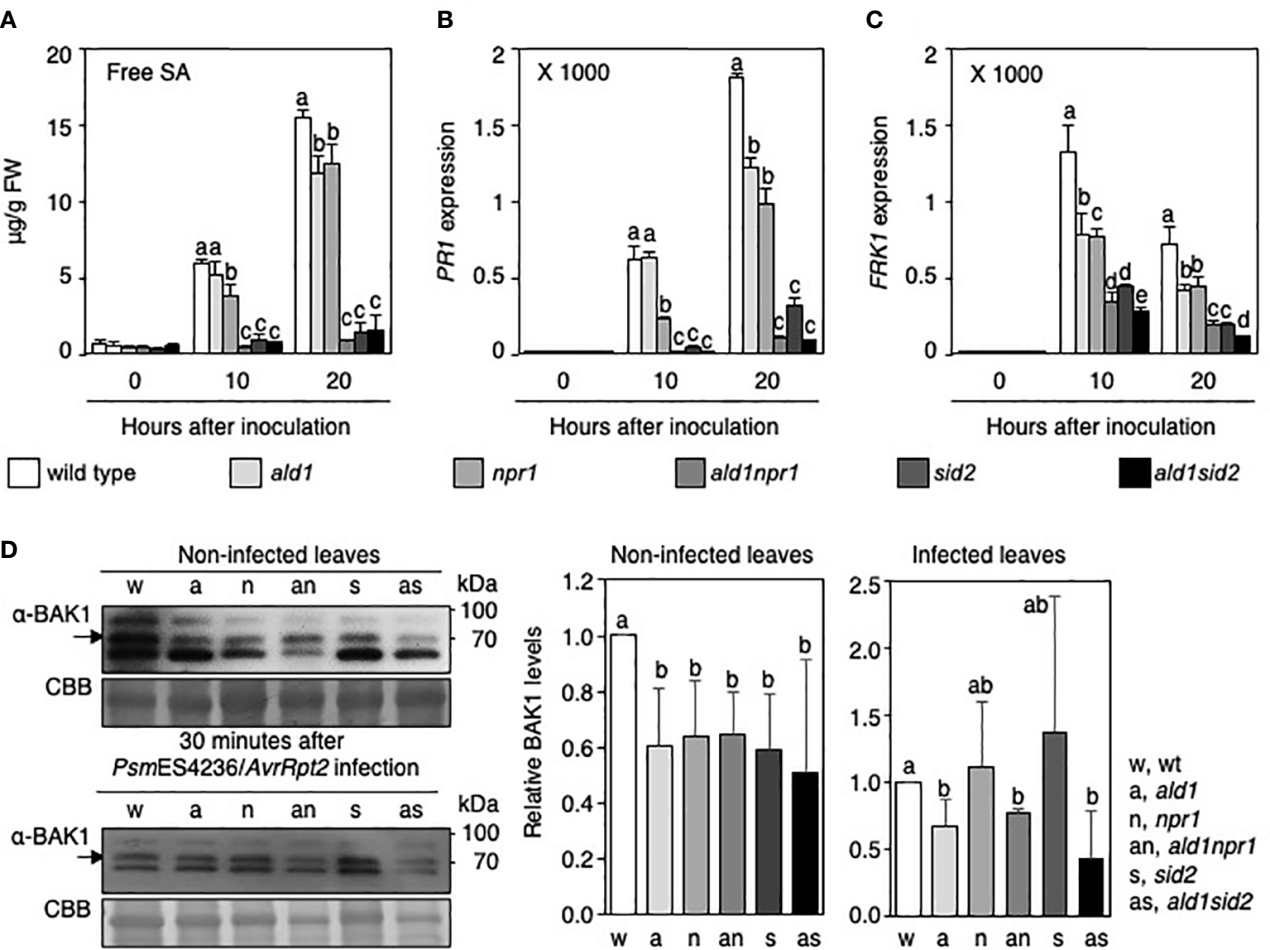
ALD1, NPR1, and SID2 are necessary to fully express RPS2-mediated immunity against an avirulent *Pseudomonas* infection. **(A)** Free SA levels in the leaves of WT, *ald1*, *npr1*, *ald1npr1*, *sid2*, and *ald1sid2* plants during avirulent *Psm*ES4326/*AvrRpt2* infection. Data show averages with standard deviations from five biological replications (n = 5). **(B)** Deregulation of *PR1* transcription in *ald1npr1* and *ald1sid2* mutants after infection. *PR1* expression levels relative to an *ACTIN2* gene in non-inoculated leaves of WT plant was set to 1. The columns and bars show the average with standard deviation obtained from three biological replications (number of technical replicates = 3). **(C)** Relative level of *FRK1* mRNA in leaves of *Arabidopsis* plants infected with *Psm*ES4326/*AvrRpt2*. We normalized expression levels and presented the results mentioned above **(B)**. **(D)** Level of BAK1 in leaves of WT and mutants in the absence or presence of avirulent pathogen infection. Upper, BAK1 protein levels in leaves of non-infected *Arabidopsis* plants; lower, BAK1 levels after *Psm*ES4326/*AvrRpt2* infection. Bands not indicated are non-specific. Graphs on the right side of **D** show the normalized BAK1 levels in *Arabidopsis* at the indicated time points (average ± standard deviation, n = 3, or 4). Different letters indicate statistically significant differences (*p* < 0.05 [**D**], *p* < 0.01 [**A-C**], one-way ANOVA).

To understand the roles of ALD1, NPR1, and ICS1/SID2 in the immune response during infection with *Psm*ES4326/*AvrRpt2* in detail, we examined the strength of SA-related defenses in WT and mutants after infection. Free SA and *PR1* mRNA levels in the *ald1* mutant were lower than those in WT plant after *Psm*ES4326/*AvrRpt2* infection (**Figures 4A, B** **)**, as previously reported (Song et al., 2004b; Cecchini et al., 2015). *NPR1* mutation resulted in reduced SA accumulation, and *PR1* transcription after *Psm*ES4326/*AvrRpt2* infection at 10 h and 20 h after inoculation compared to WT plants (**Figures 4A, B** **)**. We note that the lower SA level in *npr1* versus WT differed from previous studies (using strain *Pst*DC3000/*AvrRpt2*) in which the opposite findings were reported (Delaney et al., 1995; Zhang et al., 2010; Ding et al., 2015). These studies used longer infection times and in one case a lower infection dose for the experiments, parameters that affect free SA accumulation. The SA and *PR1* mRNA levels significantly decreased in the infected leaves of the *ald1npr1* mutant after *Psm*ES4326/*AvrRpt2* infection compared with those in *ald1* and *npr1* mutants (**Figures 4A, B** **)**. On the other hand, both SA and *PR1* mRNA did not accumulate to significant levels in *sid2* and *ald1sid2* mutants after infection (**Figures 4A, B** **)**, which is consistent with ICS1/SID2 being directly involved in SA biosynthesis (Wildermuth et al., 2001; Garcion et al., 2008). These results suggest that ALD1 acts additively with NPR1 to execute requisite defenses in RPS2-mediated ETI.

### Accumulation of BAK1 and *FRK1* expression requires ALD1, NPR1, and ICS1/SID2 in avirulent *Psm*ES4326/*AvrRpt2*-infected *Arabidopsis*

An interconnection between MAPK signaling and SA-related defense was well-described in *Arabidopsis* infected with various derivatives of *P. syringae* (Tsuda et al., 2013), and ALD1, NPR1 and ICS1 are also known to participate in basal defense responses (Laluk et al., 2011; Yi et al., 2014; 2015; Cecchini et al., 2015). Based on this knowledge, it seemed possible that additional mutation of ALD1 in *npr1* or *sid2* mutant background could weaken early defense against *Psm*ES4326/*AvrRpt2* infection. To address this, we examined representative basal defense responses in leaves of WT, single, and double mutants infected with *Psm*ES4326/*AvrRpt2*. *FRK1* mRNA levels were severely reduced in *ald1npr1* and *ald1sid2* at 10 and 20 h after *Psm*ES4326/*AvrRpt2* infection compared with *ald1*, *npr1* and *sid2*, respectively (**Figure 4C**). Compared with *FRK1* transcript levels in WT and mutants under infection of an attenuated *P. syringae* strain (scale of Y-axis was up to 12) (**Figure 2A**), infection of avirulent strain *Psm*ES4326/*AvrRpt2* could actively trigger the transcription of *FRK1* (scale of Y-axis was up to 2,000). The results support prior observations that the expression of MAMP-responsive defense responses is intensified during ETI in *Arabidopsis* (Tsuda et al., 2013; Ngou et al., 2021; Yuan et al., 2021). Furthermore, BRI1-ASSOCIATED RECEPTOR KINASE1 (BAK1) protein levels decreased in the mutants compared with that in WT plant in the absence of *Pseudomonas* infection (**Figure 4D**). After infection, however, BAK1 proteins accumulated to the WT level in *npr1* and *sid2* mutants, and BAK1 levels in the double mutants could not be distinguished from *npr1* and *sid2* mutants (**Figure 4D**). Only the ALD1 mutation adversely affected the level of BAK1 protein during *Psm*ES4326/*AvrRpt2* infection (**Figure 4D**). This indicates that ALD1 is involved in controlling MAMP-receptor levels before infection as described previously (Cecchini et al., 2015) as well as during infection. Taken together, these results suggest that the reduction of SA-related defense and early defense responses might be the cause of the high susceptibility to *Pseudomonas* carrying *AvrRpt2* of the *ald1sid2* and *ald1npr1* double mutants.

### Both NPR1 and ICS1/SID2 are required for producing active petiole exudates capable of inhibiting bacterial growth in *Arabidopsis*

**Figure 5:**
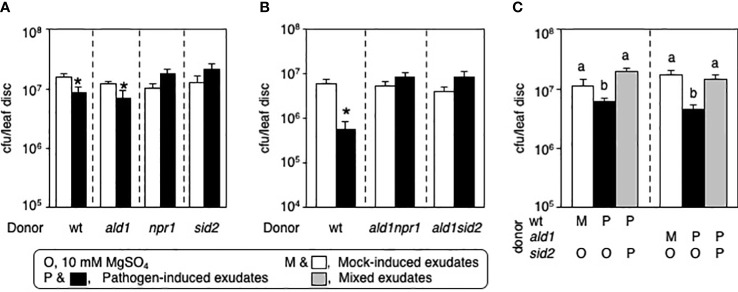
NPR1 and SID2 are needed to make active exudates capable of inducing disease resistance in plants. **(A)** Bacterial growth in the recipient WT plants pre-immunized with mock-induced exudates (MEX, white boxes) and pathogen-induced exudates (PEX, black boxes) from WT, *ald1*, *npr1*, and *sid2* plants. **(B)** Biological activity of PEXs collected from *ald1npr1* and *ald1sid2* double mutants. **(C)** Inhibitory effect of PEX from *sid2* mutant on active PEXs collected from WT and *ald1* infected with SAR-inducing *Psm*ES4326/*AvrRpt2*. Mixed exudates were pre-applied into the leaves of recipient WT plants prior to challenge-inoculation. m, mock-induced exudates; p, pathogen-induced exudates. Data represent the averages with standard errors. The asterisks (*p* < 0.05, two-tailed Student’s t-test, n = 8) and different letters (*p* < 0.05, one-way ANOVA, n = 8) indicate statistically significant differences. The experiments were repeated at least twice with similar results.

PTI and ETI that occur in local tissues can initiate SAR by inducing the accumulation of mobile signals. Application of *Psm*ES4326/*AvrRpt2*-induced petiole exudate (PEX) from WT and exudate from ALD1-overexpressing plants to recipient WT plants confers disease resistance against virulent *P. syringae* infection (Jung et al., 2009; Cecchini et al., 2015). Although it is well-known that *npr1* and *sid2* mutants have a defect in systemic resistance, the role of NPR1 and ICS1/SID2 in the biosynthesis of mobile SAR signal compounds in *Arabidopsis* is still not known. Therefore, we introduced PEXs collected from WT, *ald1*, *npr1*, and *sid2* infected with *Psm*ES4326/*AvrRpt2* into leaves of recipient WT plants 2 days prior to challenge-inoculation with a virulent *P. syringae* strain (*Psm*ES4326). PEXs from WT and *ald1* mutant successfully inhibit *Psm*ES4326 growth in the leaves of recipient WT plants (**Figure 5A**). However, neither mock-induced exudates (MEX) nor PEXs from *npr1* and *sid2* mutants could reduce bacterial growth in the recipient WT leaves after subsequent *Psm*ES4326 infection (**Figure 5A**). *ald1npr1* and *ald1sid2* mutants displaying increased susceptibility to *Pseudomonas* carrying *AvrRpt2* also failed to produce active PEXs (**Figure 5B**). These results suggest that NPR1 and ICS1/SID2 are required for producing active PEX in *Arabidopsis.* We note that SA production/transduction in *Arabidopsis* could be needed to enable tissue to produce enough mobile signals to confer disease resistance or SA might be needed as a component of the exudate, even if it is not the only mobile signal in the exudate. Our results also show a requirement for the basal level of SA before pathogen infection and accumulation of SA in local infected leaves.

In a different scenario, it could also be possible that an inactive PEX can inhibit the activity of an active PEXs. To examine if inactive *sid2*-PEX can suppress active PEX’s activity, we produced the mixed PEXs collected from different plants and introduced them into recipient WT plants before subsequent pathogen inoculation. Remarkably, the WT-PEX and *ald1*-PEX lost their immune-inducing activity in the presence of PEX from the *sid2* mutant (**Figure 5C**). This result indicates that an unidentified metabolite(s) increased in the infected *sid2* plant, or a *Pseudomonas*-derived compound(s) that accumulates more in the *sid2* mutant, may override the positive effects of WT-PEX and *ald1*-PEX.

## Discussion

The activities of ALD1, NPR1 and ICS1/SID2 can influence each other at the transcriptional level (Song et al., 2004b; Cecchini et al., 2015). This study showed that their functional relationship with respect to signaling output and pathogen suppression is complex. The loss of ALD1-dependent signaling does not make *npr1* and *sid2* mutants more susceptible to infection with an attenuated strain, virulent strains, and an avirulent strain of *P. syringae* carrying *AvrRpm1*. However, to fully activate the immune response to an avirulent *P. syringae* strain carrying *AvrRpt2* requires ALD1 in addition to NPR1 and ICS1/SID2. This highlights the differences in signaling requirements even with closely related *P. syringae* strains. Our working model is that under many conditions, the major role of ALD1 is to amplify the accumulation of SA. However, under very defense-demanding conditions ALD1 provides an additional defense function to provide robust disease resistance.

In most infections that we studied, each defense module relying on ALD1, NPR1 or ICS1/SID2 can be sufficient to inhibit attenuated or virulent *P. syringae* growth; an additive effect between ALD1 and NPR1 or ICS1/SID2 is not detectable during these *Arabidopsis*-*P. syringae* interactions. Interestingly, the *ald1* mutant shows disease susceptibility that differs from WT in a manner that depends on the infection dose. When the dose is low, *ald1* shows a WT-like disease response to the attenuated strain, but higher doses elicit stronger phenotypes. A plausible explanation is that basal immunity, although reduced, is strong enough to confer disease resistance against the low dose of attenuated *P. syringae*. Possibly different proteins, such as EDS1 and PAD4, that act upstream of ICS1/SID2 and NPR1, are still active in the *ald1* mutant (Falk et al., 1999; Jirage et al., 1999) and consequently they can regulate the functions of ICS1/SID2 and NPR1. An alternative scenario is that ALD1 may be necessary for executing a vigorous immune response when *Arabidopsis* plants are exposed to a high inoculum of virulent pathogen. For another clue, the *ald1sid2* double mutant was less resistant than each single mutant when plants were inoculated with a higher concentration of a virulent *Pseudomonas* strain (OD_600_ = 0.001) (Bernsdorff et al., 2016) than was used in this study (OD_600_ = 0.0001). The previous report supports the possibility that the strength of the immune response correlates with the intensity of pathogen threat and is determined by the relationship between individual defense modules controlled by key defense proteins. This scenario can also support ALD1’s role as an amplifier of defenses (Song et al., 2004b).

ALD1 is more important in the plant defense response during pathogenesis after spray-inoculation (*i.e.* indistinguishable from that of *npr1*, *sid2*, *ald1npr1*, and *ald1sid2*) than after syringe-inoculation. This is consistent with ALD1 playing a key role in the epidermis (Jiang et al., 2021), the cell layer that first comes in contact with sprayed bacteria. Moreover, it is known that the PTI response in plant epidermal cells is critical for sensing invading pathogens and preventing pathogens’ colonization (Melotto et al., 2006; Zeng and He, 2010; Henty-Ridilla et al., 2013; Kang et al., 2014) and that ALD1 regulates levels and responsiveness of PRRs (Cecchini et al., 2015). Thus, we suggest that ALD1 can boost PTI responses in the epidermis.

Additional mutation of *ALD1* in the *sid2* mutant does not affect SA biosynthesis and *PR1* mRNA expression. However, transcription of *FRK1* during infection significantly decreases in the *ald1sid2* double mutant compared to the single mutants. These results suggest that SA- and ALD1-mediated defense molecules have an additive effect on the amplification of *FRK1* expression in plants during ETI (Tsuda et al., 2013; Bigeard et al., 2015). The reduced immunity in *ald1npr1* and *ald1sid2* mutants after avirulent *P. syringae* carrying *AvrRpt2* infection could be due to the weakened SA-related defense as well as basal defense response resulting from the simultaneous mutation of *ALD1* and *NPR1* or *ICS1*/*SID2*. Therefore, we infer that AvrRpt2-triggered immunity may depend on PTI more than AvrRpm1-dependent immunity, where there is no additive effect of such simultaneous mutations. Taken together, we propose that these three defense proteins modulate the strength of the immune response in an infection-specific manner.

Petiole exudates from infected plants contain signal molecules, some of which may be mobile and involved in systemic immunity. It may be surprising that *ald1* exudates still have biological activity, given the proposed function of ALD1-dependent metabolites Pip and NHP as systemic signals (Návarová et al., 2012; Chen et al., 2018; Hartmann et al., 2018). However, previous experiments using chimeric plants that only produced ALD1 in epidermal cells of leaves used for generating SAR signals showed that exudates from such plants lacked Pip and NHP (Jiang et al., 2021). These chimeric plants still showed robust SAR. The present results showing that *ald1* plants can produce active exudates supports the view that SAR can occur without appreciable accumulation of vascular-mobilized Pip or NHP. This is consistent with the idea that there may be multiple priming signals that together form a priming threshold that enables strong systemic resistance.

Unlike exudates from WT and *ald1*, the exudates from *npr1* and *sid2* mutants infected with *Psm*ES4326/*AvrRpt2* are biologically nonfunctional. We do not have any direct evidence to explain the inability of *npr1*- and *sid2*-exudates collected after infection to confer immunity, but one possibility is that the *npr1* and *sid2* mutants can not produce enough signal molecule(s). It is possible that, unlike in tobacco (Vernooij et al., 1994), SA-related immunity may be needed in local tissues. This could be addressed in the future using chimeric *Arabidopsis* plants. The Pip level in petiole exudates from a *sid2* mutant was reported to be similar to that from WT plant (Návarová et al., 2012), but other proposed mobile signal molecules, such as MeSA, azelaic acid, glycerol-3-phosphate, and dehydroabietinal, have not yet been quantified in PEXs from *npr1* and *sid2* mutants (Jung et al., 2009; Chanda et al., 2011; Chaturvedi et al., 2012; Dempsey and Klessig, 2012; Návarová et al., 2012). Thus, we infer that all or some of these proposed signals may not accumulate in *npr1*- and *sid2*-PEX. Alternatively, it is also conceivable that both mutants impair the ability to mobilize signals out of the leaves into the exudate.

Another plausible explanation for the non-functionality of the *npr1* and *sid2* exudates may be related to systemic induced susceptibility (SIS, Cui et al., 2005; Haney et al., 2018). This is a phenomenon wherein plants inoculated on their lower leaves with a strain that produces the secreted toxin coronatine (a mimic of jasmonic acid; Mittal and Davis, 1995; Bender et al., 1999; Katsir et al., 2008) become more susceptible to infection of the upper leaves (Cui et al., 2005). PEXs from the hypersusceptible *npr1* and *sid2* mutants may contain a high level of coronatine and/or plant metabolite(s) that suppress systemic resistance in plants. This may occur because *Psm*ES4326/*AvrRpt2* is more virulent in these mutants and more secreted coronatine may end up in the *npr1* and *sid2* exudates. The fact that mixed exudates between active exudates from WT and *ald1* and PEX from the *sid2* mutant can not suppress bacterial growth in the recipient WT plants strongly supports this last explanation. We note that coronatine inhibits SA-dependent defenses by disturbing SA accumulation, resulting in local and systemic susceptibility (Geng et al., 2012; Zheng et al., 2012). Therefore, coronatine is a strong bacterial-derived candidate that accumulates in s*id2*-PEX. It is also possible that the jasmonic acid accumulated in the leaves of *sid2* donor plants inoculated with *P. syringae* carrying *AvrRpt2* (Liu et al., 2016) inhibits the PEX-induced resistance in recipient WT plants by blocking the activation of SA-related defenses (Koornneef and Pieterse, 2008).

In conclusion, to control bacterial growth, ALD1 acts independently or together with NPR1 or ICS1/SID2 as a defense signal amplifier, depending on the type of *Arabidopsis*-*Pseudomonas* interaction. Both defense modules are required to confer maximal immunity to infection by an avirulent pathogen that triggers resistance *via* perception of the AvrRpt2 bacterial effector.

## Data availability statement

The original contributions presented in the study are included in the article/supplementary material. Further inquiries can be directed to the corresponding author.

## Author contributions

HWJ and JG conceived and designed the experiments. HWJ, S-JY, HJC, and SWN performed the experiments. HWJ, JG, and NC analyzed and interpreted data and wrote the paper. All authors contributed to the article and approved the submitted version.

## Funding

This work was supported by Basic Science Research Program through the National Research Foundation of Korea funded by the Ministry of Education (2020R1A6A1A03047729) and the Green Fusion Technology Graduate School Program of the Ministry of Environment to HJ, a grant from NSF (NSFIOS1456904) to JG, and grants from ANPCYT and CONICET (PICT-2017-0589, PICT-2020-0483, and PIP_2021-2023) to NC.

## Conflict of interest

The authors declare that the research was conducted in the absence of any commercial or financial relationships that could be construed as a potential conflict of interest.

## Publisher’s note

All claims expressed in this article are solely those of the authors and do not necessarily represent those of their affiliated organizations, or those of the publisher, the editors and the reviewers. Any product that may be evaluated in this article, or claim that may be made by its manufacturer, is not guaranteed or endorsed by the publisher.
